# Differences in somatostatin receptor subtype expression in patients with acromegaly: new directions for targeted therapy?

**DOI:** 10.1007/s42000-021-00327-w

**Published:** 2021-10-21

**Authors:** Lena Rass, Amir-Hossein Rahvar, Jakob Matschke, Wolfgang Saeger, Thomas Renné, Jens Aberle, Jörg Flitsch, Roman Rotermund

**Affiliations:** 1 Department of Neurosurgery, University Medical Center Hamburg-Eppendorf, Martinistr. 52, 20246 Hamburg, Germany; 2 III. Department of Medicine, University Medical Center Hamburg-Eppendorf, Hamburg, Germany; 3 Institute of Neuropathology, University Medical Center Hamburg-Eppendorf, Hamburg, Germany; 4 Institute of Clinical Chemistry and Laboratory Medicine, University Medical Center Hamburg-Eppendorf, Hamburg, Germany

**Keywords:** Acromegaly, Somatostatin receptor subtypes 2a and 5, Immunohistochemistry, Monoclonal antibodies, Somatostatin analogs

## Abstract

**Purpose:**

To analyze the expression of somatostatin receptor (SSTR)2a and 5 by immunohistochemistry (IHC) in surgically resected somatotrophic pituitary adenomas and to associate expression rates with tumor size and clinical, biochemical, and histological parameters and response to somatostatin analog (SA) therapy.

**Methods:**

Forty-three microsurgically treated patients with histopathologically proven growth hormone (GH)–producing pituitary adenoma were included (WHO 2017). SSTR subtype expression was analyzed in adenoma tissues using monoclonal antibodies (Abcam, SSTR2a-UMB1, SSTR5-UMB4). Expression rates were classified as low (≤ 20% staining positivity), moderate (21–50%), and high (> 50%). Furthermore, biochemical parameters such as human growth hormone (hGH) and insulin-like growth factor-1 (IGF-1) levels were measured and clinical, biochemical, radiological, and histological data were evaluated.

**Results:**

Of the 43 patients included in this study, 28 were female and 15 were male. The median age was 52 years (range 17–72 years). The median tumor size was 1.2 cm (range: 0.13–3.93 cm). All resected tumors showed positivity for somatotrophic hormone (STH). In all tissue samples, SSTR2a signal expression was detectable in immunohistochemistry, while only 39 samples were positive for SSTR5. Thirty-six samples had a high expression of SSTR2a, while three had a moderate and four a low SSTR2a signal. In comparison, SSTR5 signal was high in 26 out of 43 samples, while seven adenomas showed a moderate and six cases a low expression rate of SSTR5. The median IGF-1 was 714.2 µg/l and the median GH 19.6 mU/l (= 6.53 µg/l). The present study indicates that there is no significant relationship between the expression rates of receptor subtypes and the parameters we analyzed. However, our study revealed that smaller adenomas have a lower baseline GH level (*p* = 0.015),

**Conclusion:**

IHC with monoclonal antibodies appears to be a suitable method to determine the expression rates of SSTR2a and 5 at protein levels, as it is not possible to draw conclusions regarding receptor subtypes solely on the basis of the parameters analyzed.

## Introduction

Acromegaly is a rare endocrinological disease caused by an excess of growth hormone (GH), also known as somatotropin. Predominantly, it is related to a benign GH-secreting tumor of the anterior pituitary gland [1]. Very rarely, malignant pituitary tumors lead to acromegaly. Disease prevalence is approximately 40–130 per million, and its annual incidence is estimated at three to four new cases per million individuals [2, 3]. The gender distribution is equal [4].

The therapy of pituitary adenomas is currently based on three different strategies, including transsphenoidal surgery (TSS), pharmacotherapy, and radiotherapy. TSS is the first-line treatment for patients with pituitary adenoma and acromegaly [5]. Full-remission rates up to 70% are reported in the current literature, using endoscopic and microscopic TSS, respectively [6, 7]. In all patients not suitable for surgery or in whom the tumor tissue could not be completely removed, first-generation long-acting somatostatin analogs (LA-SSAs octreotide and lanreotide) are regarded as the primary choice of pharmacotherapy [5]. In patients with resistance to first-generation somatostatin receptor ligands (SRL), pegvisomant, a GH receptor antagonist, and pasireotide, a second generation SRL, may be used [8, 9]. Current meta-analyses demonstrate that the response rate to SSAs is about 56% for GH and 55% for insulin-like growth factor-1 (IGF-1) normalization. In treatment-naïve patients, GH normalization can be achieved in about 40% [10, 11].

Besides their application in incomplete tumor resection, SSAs can be used for preoperative size reduction of pituitary adenomas, as these drugs induce a volume reduction of more than 20% in up to 75% of cases [10, 11].

Somatostatin (SST), in its biologically active forms SST14 and SST28, inhibits the secretion of GH [11]. The effect of SST is mediated by five distinct G-protein-coupled somatostatin receptors (SSTR), namely, SSTR1–5. SSTR2 und SSTR5 are the most prominent in somatotropic adenomas according to the current literature [10, 12–14]. Octreotide has the highest affinity to SSTR2 and a low affinity to SSTR3 and SSTR5. Lanreotide also shows the strongest affinity to SSTR2, followed by SSTR1, and shows low affinity to SSTR3 and SSTR5 [13, 15]. Pasireotide, however, has a high affinity to SSTR2 and 3 as well as to 5, and a moderate affinity to SSTR1 [14–17]. Due to these diverging affinities to SSTR subtypes, histopathological analysis of SSTRs may prove diagnostically significant.

In this study, we examined the expression rates of SSTR2a and 5 in surgically resected somatotropic pituitary adenomas by assessing signal intensity of monoclonal antibody-binding to cognate receptors. Immunohistochemistry (IHC) data were set in relation to tumor size, clinical chemistry, and response to SA therapy.

## Material and methods

### Patients/tumor samples

Forty-three patients, diagnosed with acromegaly, were included in this study. All patients underwent microscopic TSS for biochemical-proven acromegaly in our Department of Neurosurgery, University Medical Center Hamburg-Eppendorf, Germany, between July 2018 and June 2019 (Table 1). The diagnosis of acromegaly was made according to the current guidelines on acromegaly of the Endocrine Society. The guidelines recommend performing IGF-1 measurement in the presence of typical clinical characteristics and/or a pituitary tumor. Patients with an increased or suspect IGF-1 level should be subjected to an oral glucose tolerance test, which, in the case of inadequate suppression of GH plasma concentration levels, will confirm the diagnosis [5]. Finally, diagnosis was made by histopathological analysis of the resected tumor tissue samples (WHO classification 2017). Fourteen tumor samples revealed prolactin co-secretion determined by IHC and increased prolactin levels.

**Table 1:** Characteristics of the cohort

	Value label	*N*
Gender	Male	15 (34.88%)
	Female	28 (65.12%)
Tumor size	Microadenoma (< 1 cm)	18 (41.86%)
	Macroadenoma (≥ 1 cm)	25 (58.14%)
Histological classification	Densely granulated	24 (55.81%)
	Sparsely granulated	19 (44.19%)
Tumor classification	GH-PRL	14 (32.56%)
	GH	29 (67.44%)
Ki67	≤ 3%	28 (65.12%)
	> 3%	15 (34.88%)
Invasiveness	Invasive	7 (16.12%)
	Non-invasive	36 (83.72%)

The tumor size was measured on the basis of an MRI, while invasion behavior was determined intraoperatively, as well as by imaging, and finally by histopathologic examination of separately submitted dura specimens and/or sphenoid mucosa. Further patient data, such as age and gender, were assessed.

### Immunohistochemistry

The preparation of the tumor specimens was carried out according to standardized laboratory procedures established by the Institute for Neuropathology, University Medical Center Hamburg-Eppendorf. In brief, the intraoperatively obtained samples were fixed in a 4% buffered formalin solution at room temperature, followed by embedding in paraffin and cutting these blocks into 2-4 µm thick sections.

The immunohistochemical procedure to determine the expression rates of SSTR2a and SSTR5 was performed by using two monoclonal antibodies (Abcam, SSTR2a–UMB1 — dilution 1:1000 and SSTR5–UMB4 — dilution 1:200) with the automated Ventana BenchMark XT as specified by the manufacturer.

**Fig. 1:**
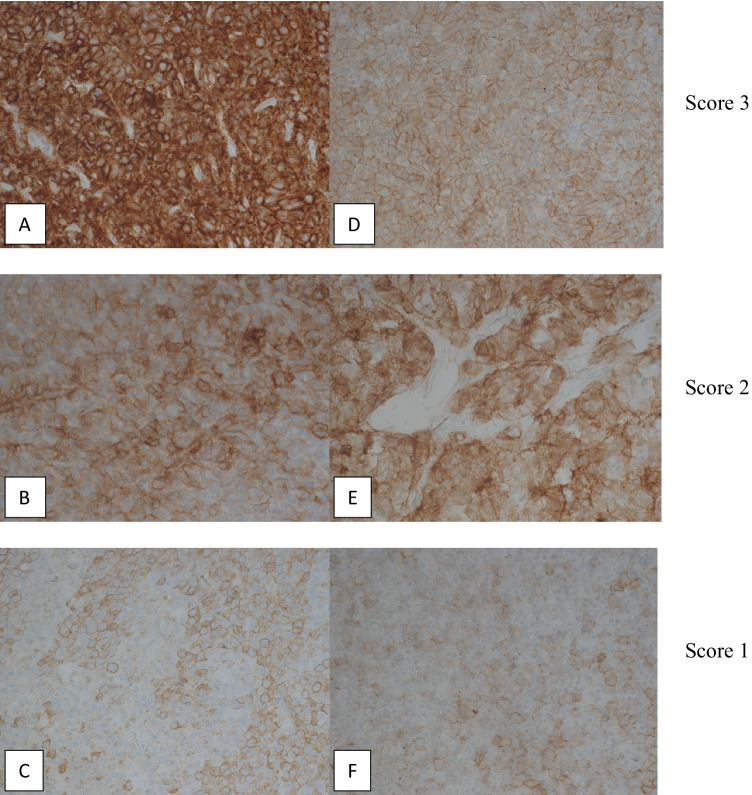
Different immunohistochemical expression levels of SSTR2a (A–C, magnification 440 ×) and SSTR5 (D–F, magnification 440 ×) in somatotrophic pituitary adenomas. Only cell membrane staining was regarded as positive and staining intensity was not considered

To assess the staining results of the IHC and thus the expression rate of SSTR2a and SSTR5, we classified the pituitary tumors into four groups in terms of their percentage of immunoreactive cells. Only cell membrane staining was considered positive. No proof of positive-stained cells corresponds to score 0. Up to 20% of immunoreactive cells correlates to score 1 (+ /low), 21–50% positive-stained cells (+ + /moderate) to score 2, and more than 50% positive cells (+ +  + /high) to score 3 (Table 2, Fig. 1). In our study, we did not consider the intensity of the staining.

**Table 2:** Immunoreactivity score based on percentage of positive-stained cells and expression of SSTR2a and SSTR5 in 43 somatotrophic pituitary adenomas

Immunoreactivity score (IRS)	Positive-stained cells (%)	SSTR2a — *N*	SSTR5 — *N*
Negative ( −)	0	0 (0%)	4 (9.30%)
Low ( +)	≤ 20	4 (9.30%)	6 (13.95%)
Moderate (+ +)	21–50	3 (6.89%)	7 (16.28%)
High (+ + +)	> 50	36 (83.72%)	26 (60.47%)

The IHC evaluation was performed by a single pathologist (W.S.) blinded to the clinical data.

### Histological classification

**Fig. 2:**
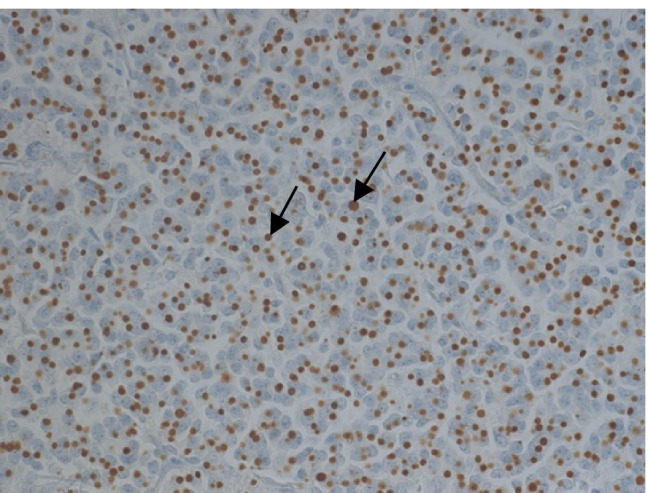
Immunohistochemical staining for cytokeratin (CAM5.2). Fibrous bodies as a feature of SG adenomas shown by arrows. Magnification 440 ×

Classification of somatotrophic pituitary adenomas into densely granulated (DG) and sparsely granulated (SG) tumors was performed using IHC and anti-cytokeratin antibodies (CAM5.2). Immunohistochemically detected fibrous bodies, which are keratin-positive, small, spherical cytoplasmic inclusions, are classified as SG tumors (Fig. 2). Accordingly, the absence of fibrous bodies characterizes DG adenomas.

### IGF-1 and hGH measurement

IGF-1 and human growth hormone (hGH) in patient serum were determined using the Siemens Healthineers IMMULITE 2000XPi solid phase-chemiluminescence immunoassay according to the manufacturer’s instructions at the Institute of Clinical Chemistry and Laboratory Medicine. The Siemens hGH and IGF-1 Sandwich-ELISA tests are based on monoclonal murine-anti-hGH and anti-IGH-1 antibodies, respectively, coupled to beads. Reference range for hGH < 15 mU/l (conversion factor mU/l-µg/l 0.333) and for IGF-1 gender- and age-adjusted reference ranges were used. Measurements were taken prior to surgery, as well as on the first and third postoperative days. Postsurgical GH serum concentrations of < 0.1 µg/l (reported GH nadir without oral glucose tolerance test) indicate disease remission.

### Ki-67%

**Fig. 3:**
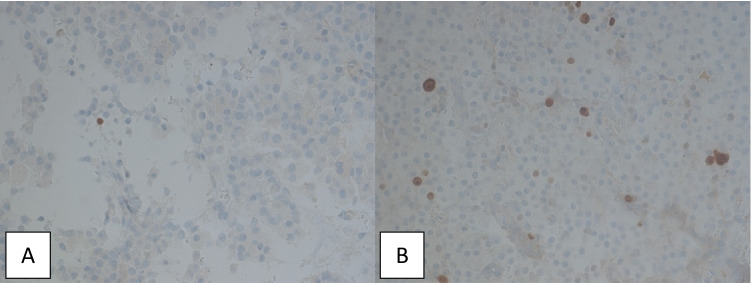
Immunohistochemical detection of proliferation marker Ki-67 in formalin fixed, paraffin embedded pituitary adenoma samples. Assessment was based on the percentage of positively stained cells. Less than 3% indicates no increased proliferation rate (A, magnification 440 ×). More than 3% suggests an increased proliferation rate (B, magnification 440 ×)

The proliferation marker Ki-67, which is expressed exclusively on the surface of dividing cells, was determined immunohistochemically to evaluate the proliferation rate of the formalin fixed, paraffin embedded tumor samples. The antibody used in our study for detection was MIB-1 (Neo Markers, RM-9106-S, dilution 1:1000). The IHC was conducted with the automated Ventana Benchmark XT staining system, based on the manufacturers’ protocols. The evaluation of the proliferation rate was based on the percentage of positively stained cells. Less than 3% of positively stained cells correspond to a regular not significantly increased proliferation rate of somatotropic tumors, whereas more than 3% of positive cells indicate a significantly increased occurrence of dividing cells (Fig. 3).

### Statistical analysis

All statistical analyses were performed using the statistical software “IBM Corp. Released 2020. IBM SPSS Statistics for Windows, Version 27.0. Armonk, NY: IBM Corp.”

Qualitative data are presented as absolute and relative (%) frequencies, while quantitative data are presented as median (min, max). Spearman’s rho test was performed to determine whether the expression of SSTR2a and SSTR5 has an influence on the response to pharmacotherapy, measured by the IGF-1 (ULN), in patients pretreated with SSA. Using linear regression analysis with preoperative IGF-1 level and GH level as dependent variables, respectively, adjusted for age and gender, their relationship with parameters such as tumor size, histology, invasion behavior, and expression rates of SSTR2a and SSTR5 was assessed. For the analysis of correlations between the SSTR expression rates and nominal variables, the Fisher exact test was performed.

A *P*-value of < 0.05 was considered a statistically significant result.

## Results

### Patients

The tumor samples that were histologically examined were taken from 43 patients of whom 28 were female and 15 were male. The median age at time of surgery was 52 years (range 17–72 years). Macroadenomas, i.e., tumors larger than or equal to 1 cm, were detected in 25 cases, while microadenomas (< 1 cm) were found in 18 patients. The median tumor size was 1.2 cm (range 0.13–3.93 cm). The median of presurgically determined growth-hormone level was 19.6 mU/l (range 4.8–150 mU/l), while the median baseline IGF-1 level was 714.2 µg/l (range 139.4–1122 µg/l) and the median presurgical IGF-1 level (/ULN) was 3.12 µg/l (range 0.63–5.01 µg/l).

### Pretreatment

Eight out of the 43 patients underwent a surgical intervention beforehand, of whom four received pharmacotherapy afterwards, which was maintained up to the next operation. One of them had combined therapy with cabergoline (dopamine D_2_-receptor agonist) and lanreotide (a SSA). The other three received octreotide (SSA). Two patients without previous surgical intervention received cabergoline, and another octreotide.

There was no significant influence of SSTR2a on the effect of pharmacotherapy with octreotide or lanreotide as assessed by age- and gender-adjusted IGF-1 concentration level after pharmacotherapy (*ρ*=-0.354, *p* = 0.559), while a more significant correlation with regard to SSTR5 was observed (*ρ*=-0.738, *p* = 0.155). However, there was just one patient who reached an age- and gender-normalized IGF-1 level under medical therapy (Table 3).

**Table 3:** SSTR 2a/5 expression and presurgical IGF-1 levels (ULN) in patients with prior SSA treatment

Gender	Previous surgery	Medication	Dose	Duration of intake	SSTR 2a	SSTR 5	IGF-1 (µg/l)/ULN
Male	Yes	Octreotide	10 mg/m	01/13–05/15 04/17–07/18	+ + +	+ + +	0.63
Female	No	Octreotide	10 mg/m	> 4 month	+ + +	+	3.78
Female	Yes	Cabergoline + Lanreotide	0.5 mg/w 120 mg/m	2015–08/18	+ + +	+ + +	1.69
Female	Yes	Octreotide	40 mg/m	06/12–08/18	+ + +	+ +	1.67
Female	Yes	Octreotide	40 mg/m	02/17–09/18	+ +	+ +	2.41

### IGF-1/hGH

In terms of presurgical IGF-1 levels, no statistically significant relationship was found regarding tumor invasion, tumor size, or expression rates of SSTR2a and SSTR5 (Table 5).

As expected, a smaller tumor size was associated with lower GH levels (*P* = 0.015, Table 4). Out of 15 patients with microadenomas in whom a presurgical GH measurement was performed, 11 (73.33%) had GH values below 5 µg/l. In contrast, among patients with macroadenomas, only two out of 20 (10%) had a level of less than 5 µg/l.

**Table 4:** Linear regression analysis adjusted for age and gender. Presurgical GH level as dependent variable

Parameter	*P* value	*B*	Std. error
Age	0.578	− 0.101	0.180
Gender	0.501	− 3.016	4.414
Histology	0.102	− 8.222	4.840
Invasiveness	0.116	− 10.313	6.322
Average tumor size (cm)	0.015**	7.799	2.972
SSTR2a + + +	0.702	− 2.855	7.369
SSTR2a + +	0.493	− 7.184	10.316
*SSTR2a +	0^a^		
SSTR5 + + +	0.531	− 5.187	8.156
SSTR5 + +	0.542	− 5.175	8.357
SSTR5 +	0.738	2.845	8.398
*SSTR5 −	0^a^		

^a^This parameter is set to zero because it is redundant

^*^Reference category; ** P-value of < 0.05 was considered statistically significant

### Remission

After surgery, 20 of 42 patients (47.6%) showed a GH level of < 1 µg/l at the time of the second measurement (day 3). In seven of these cases, the value was below < 0.4 µg/l. In contrast, eight patients (19.1%) had a GH level of > 3.5 µg/l. Seven of them were macroadenomas (87.5%), of which two showed invasive growth. The GH level of the remaining 14 cases was between 1 µg/l and 3.5 µg/l, of which nine were below 2 µg/l. In one case, no results were present for the second measurement.

Forty patients showed a decrease in IGF-1 level, of whom seven (17.5%) had already reached an age-normalized IGF-1 serum concentration at the second postoperative measurement (day 3).

### Pituitary function

In 38 cases (88%), there was no insufficiency of the pituitary axes after surgery. In most cases with postsurgical pituitary deficits, there was already a loss of function before intervention (80%; 4/5). Three patients showed insufficiency of the pituitary gland presurgically, which recovered afterwards. Depending on each case, functional disorders such as corticotropic, thyrotropic, gonadotropic insufficiencies, and/or central diabetes insipidus were present, and an appropriate therapy and follow-ups were initiated.

### Histology

All 43 removed adenoma tissue samples used in our study stained immunohistochemically positive for somatotrophic hormone (STH), of which 14 were also positive for prolactin. A histological classification into DG and SG adenomas was made (DG 55.8%, SG 44.19%). An increased proliferation rate (Ki67 > 3%) was observed in 15 specimens (34.9%) (Table 1).

Expression of SSTR2a was detected in all tumor specimens, whereas SSTR5 expression was absent in four samples.

**Fig. 4:**
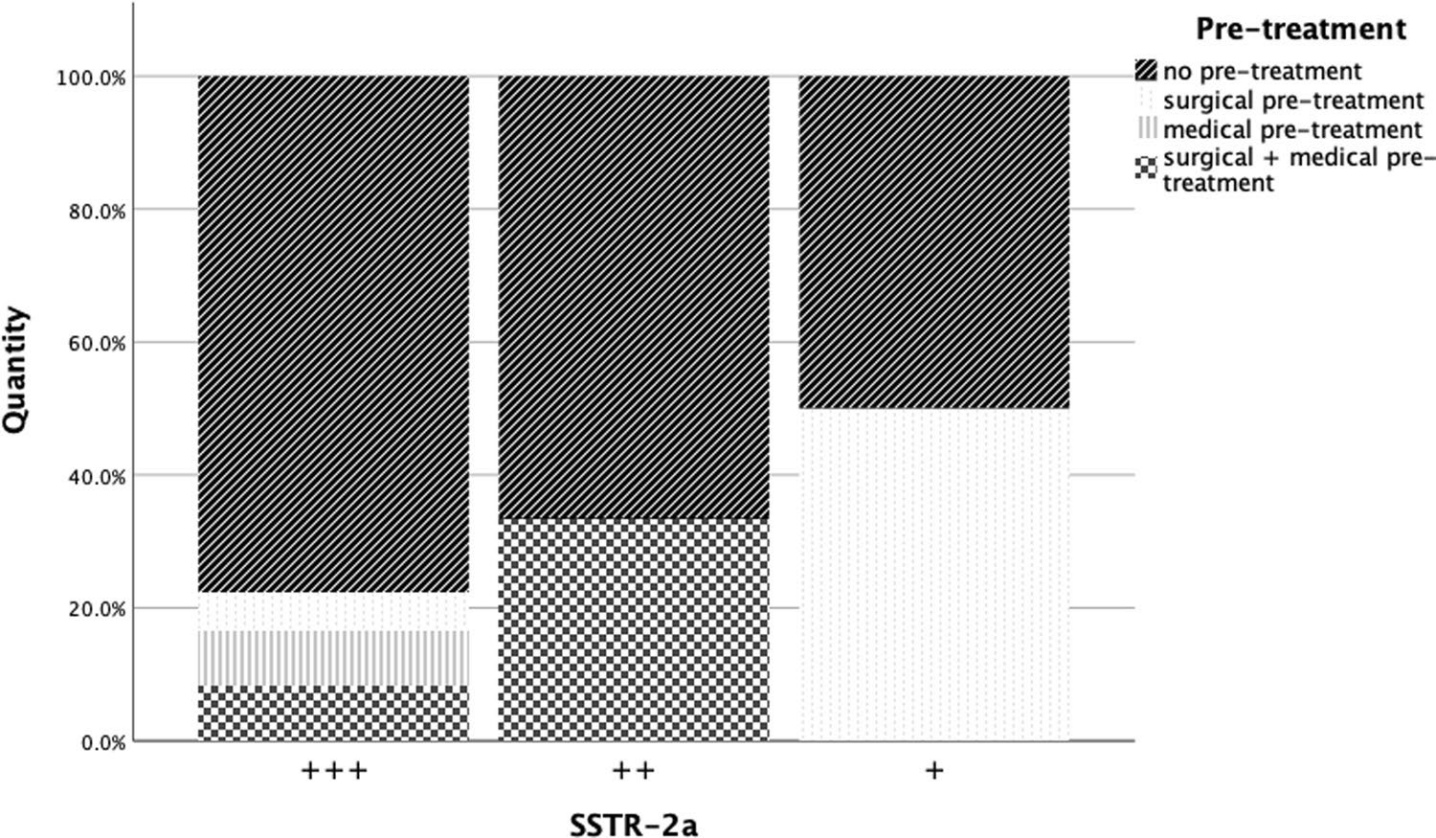
Pre-treatment with respect to SSTR2a

**Fig. 5:**
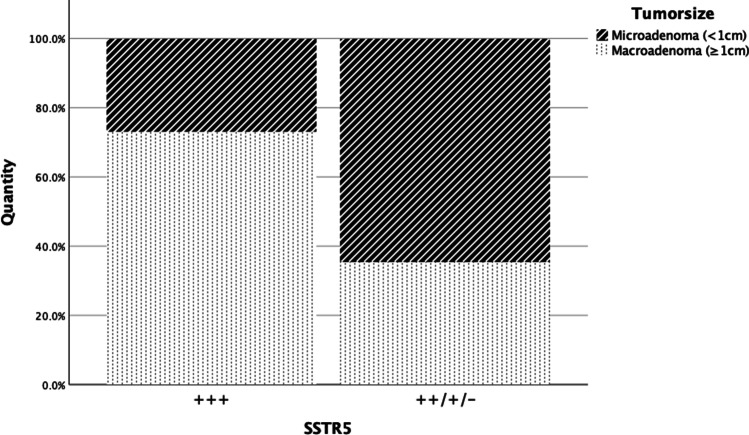
Tumor size with respect to SSTR5

High expression rates for SSTR2a with more than 50% immunoreactive cells were found in 36 cases; in three samples, the number of positive cells was between 21 and 50%, which represents a moderate expression rate, and four had a low SSTR2a signal with only up to 20% positivity. Lower expression rates of SSTR2a were often associated with previous operations and occurred more frequently with SG tumors (Figs. 4 and 5). Out of seven tissue samples which showed a moderate or low expression rate with respect to SSTR2a, five were SG (71.4%). However, these characteristics were also present in samples with strong expression. Out of seven tumors that grew invasively, six (85.7%) had a high expression rate of SSTR2a.

**Fig. 6:**
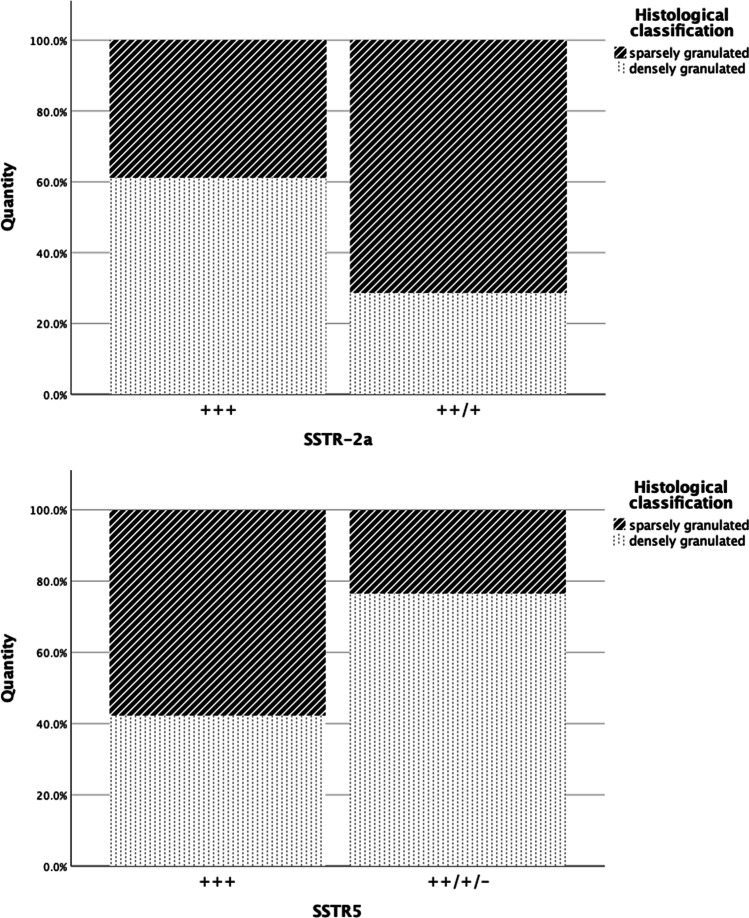
Histological classification with respect to SSTR2a/SSTR5

In comparison, 26 out of 43 adenomas showed more than 50% positive-stained cells (high expression) regarding SSTR5, while seven samples showed moderate and six a low expression rate. With regard to SSTR5, lower or no expression was more frequent with DG tumors (76.5%, 13/17, Fig. 5), and DG were also positive for prolactin in 9/14 cases (64.3%). SG tumors with no or lower expression of SSTR5 were all, except for one, associated with prior surgery (75%, 3/4). Furthermore, a low level or no expression of SSTR5 was mostly associated with adenoma size smaller than 1 cm (70%, 7/10, Fig. 6).

With regard to prolactin co-secreting adenomas, 13 showed high expression of SSTR2a, and only one had a low SSTR2a expression rate. High expression of SSTR5, however, was seen in only six GH/PRL samples, while three showed moderate, three low, and two no expression of SSTR5.

## Discussion

In this study, we investigated expression of SSTR2a and SSTR5 in surgically resected somatotropic adenomas of the pituitary gland.

The expression of somatostatin receptors in different tissues has been ascertained in other studies, mostly by RT-PCR, in situ hybridization, scintigraphy, receptor autoradiography, or IHC with polyclonal antibodies [18–22]. In our case, we used IHC with two monoclonal antibodies in an automated staining procedure. These specific antibodies have been shown to be sensitive and reliable in previous studies, and they show no cross-reaction with proteins other than the targeted ones, as can occur with polyclonal antibodies [23, 24]. As far as the automatic procedure is concerned, according to recent data, it seems to be an advantageous method for determining SSTR expression rates in tumor tissues [25].

Whereas several studies describe receptor expression rates at mRNA level, we performed receptor-protein analysis by using IHC [19, 26–31]. Some studies show a correlation between these two methods [29, 31, 32]. However, in this respect, the various studies are still ambiguous, which is probably due to the process that takes place to turn mRNA into a protein [32–35].

An advantage of IHC compared to other methods is its ability to provide information on the sub-cellular localization of SSTR [36, 37]. In our case, as expected for G-protein-coupled receptors, signals were mainly located on the cell membrane. Additionally, an immunoreactive score can be generated, which provides information about the SSTR expression rate (Table 2). In comparison to other studies, we did not consider the intensity of the staining, but concentrated exclusively on the percentage of immunoreactive cells to avoid a subjective assessment of the staining intensity [23, 28, 35, 36, 38, 39]).

The occurrence of SSTR2a and SSTR5 in all or at least the majority of somatotropic adenomas was reported in earlier studies [25–27, 35, 40, 41]. According to the literature, pharmacotherapy with somatostatin analogs to inhibit hormone secretion and reduce cell proliferation seems to be associated with the presence of mainly subtypes SSTR2 and SSTR5 [10, 12–14].

Our data showed that the majority of GH-secreting tumors studied had a high expression of both SSTR2a and SSTR5 (83.72% and 60.47% respectively), which is also consistent with previous publications [18, 25].

In contrast to the surveys of Thodou et al. and Jaquet et al., SSTR2a (100%) occurred more frequently compared to SSTR5 (90.7%) [18, 27]. Other authors, however, confirmed our result [25, 30, 35]. One reason for these differing findings may be the different methods used in the individual studies to determine the receptors’ expression.

With regard to the expression rates of SSTR2a and SSTR5, there is no significant difference between GH/PRL tumors and pure GH tumors. This is consistent with previous analyses, but differs from the research of Casarini et al., who found a higher incidence of SSTR5 in co-secreting adenomas [18, 19, 25, 27]. Unlike this result, our study showed that five out of ten tumors (50%) with no or low expression of SSTR5 are GH/PRL tumors. A further investigation of this aspect with a larger cohort is required to draw firmer conclusions regarding the therapy with pasireotide in GH/PRL tumors, a pharmaceutical substance with a high affinity to SSTR 5 [15, 17].

Contrary to our assumption, SSTR2a expression had no influence on the response to pharmacotherapy with octreotide or lanreotide measured by IGF-1 levels after long-term medication. There was a negative correlation between the gender- and age-adjusted IGF-1 levels and SSTR5 expression.

We also cannot corroborate on the basis of our study the results of Fougner et al. and Plöckinger et al. showing a possible effect of prior medical treatment on SSTR expression (mainly SSTR2a) [38, 40]. While these two research studies suggest SSTR2a reduction by presurgical SSA therapy, four out of five of our patients pretreated with SSAs exhibited high and one moderate SSTR2a expression levels.

These differing results are probably due to our limited number of five patients who received pharmacological pre-treatment with somatostatin analogs.

With regard to gender and age, there was no difference in SSTR2a and SSTR5 expression rate in our population.

In terms of SSTR2a expression, tumor size did not seem to play a significant role either. These results are in agreement with the findings of Plöckinger et al., as well as of Corbetta et al., who also failed to reveal a correlation between SSTR2 and tumor size [26, 40]. Even though our results did not show a statistically significant correlation between SSTR5 and tumor size, macroadenomas appear to have a slightly higher SSTR5 expression rate than microadenomas.

Additionally, while Corbetta et al. did not find any correlation between SSTR2 mRNA level and tumor invasiveness, our findings indicated that invasive tumors have a high expression of SSTR2a (85.71%, 6/7) [26]. This result is presumably due to our low number of invasively growing tumors.

The assumption that smaller adenomas have lower baseline GH level was confirmed in our present study (*p* = 0.015) and is in accordance with the previous report of Kaltsas et al. ([42]). Similarly to Casarini et al., however, we found no significant correlation between SSTR expression and pre-surgical GH and IGF-1 levels [19] (Tables 4 and 5).

**Table 5:** Linear regression analysis adjusted for age and gender. Presurgical IGF-1 (/ULN) level as dependent variable

Parameter	*P*** value**	*B*	Std. error
Age	0.735	− 0.005	0.013
Gender	0.330	− 0.371	0.375
Histology	0.140	− 0.605	0.400
Invasiveness	0.709	0.173	0.461
Average tumor size (cm)	0.109	0.422	0.256
SSTR2a + + +	0.204	− 0.773	0.596
SSTR2a + +	0.229	− 1.022	0.833
*SSTR2a +	0^a^		
SSTR5 + + +	0.985	− 0.013	0.669
SSTR5 + +	0.854	− 0.130	0.700
SSTR5 +	0.886	0.106	0.728
*SSTR5 −	0^a^		

^a^This parameter is set to zero because it is redundant

^*^Reference category

Looking at the GH levels at time of the second postoperative measurement, GH suppression below 1 µg/l indicates a high likelihood of complete remission [43–45]. Values below 0.4 µg/l may have an even higher predictive value for remission [43].

In contrast, GH levels > 3.5 µg/l lead to relapse, with a very high chance of up to 100%, according to the aforementioned studies [43–45]. Our study showed that cases with GH values > 3.5 µg/l after surgery were mainly macroadenomas.

Regarding the histologic classification, the ratio in our study was 55.81% (DG) to 44.19% (SG). This result is in accordance with other studies in the literature, wherein a wide range of variation can be found, the latter probably due to the different ways of categorizing such tumors [41, 46–48].

Looking at the somatostatin receptor distribution among DG and SG tumors, our analysis revealed that the majority of DG (91.67%, 22/24) had high expression of SSTR2a, while it was slightly less in SG (73.68%, 14/19), which has been confirmed in several studies [25, 28, 29, 39, 49]. In comparison, 11 DG (45.83%, 11/24) and 15 SG (78.95%, 15/19) showed a high expression rate of SSTR5. This leads to the hypothesis that DG show a higher response to medical treatment with somatostatin analogs such as octreotide, which have a high affinity to SSTR2, as supported by the analysis of Ezzat et al. In their study, DG adenomas showed a higher decrease in GH levels by inhibition of GH secretion during pharmacotherapy with octreotide than did SG adenomas [50].

Similarly to the majority of previous reports, our findings demonstrated that SG compared to DG more often occurred at younger age and had a larger volume at the time of diagnosis, which may be partly explained by the increased Ki67 in SG and young patients, as this was seen in about half of the cases in our cohort [25, 29, 41, 46, 48, 51, 52].

In terms of invasiveness, however, there was no remarkable difference between SG (4/7 cases 57.14%) and DG (3/7cases 42.86%). In this respect, there is controversy among the various authors of previous studies. While in the study of Chinezu et al. there is evidence that SG have a more invasive character, the study of Brzana et al. shows no correlation between invasiveness and the different subtypes [25, 41]. One reason for these diverse research results could be the subjective assessment of tumor size and invasiveness based on an MRI.

Regarding gender, the majority of studies, including ours, could not find any correlation [28, 46, 47]. However, the study of Mazal et al. revealed that SG are more common in women [51].

## Conclusion

Our study demonstrating that it is apparently not possible to determine receptor subtypes solely on the basis of the analysis of parameters, we determined that IHC is a useful method to ascertain the best possible treatment for each individual.

Our study demonstrates the feasibility of IHC examination of SSTR2a and SSTR5 in GH adenomas.

Today, TSS still is the first-line therapy for acromegaly. However, there are cases in which complete surgical removal of the tumor is not possible. In these cases, pharmacotherapy with somatostatin analogs is recommended. Since the response to medical treatment is to a certain extent related to the expression of SSTR in the target tissue, a reproducible method is necessary as a routine procedure for determination of SSTR expression. This is relevant in order to be able to determine which is the most effective SSA individually for each patient, since the various analogs have diverging affinities to SSTR2a and SSTR5. The technique of IHC with monoclonal antibodies, which we used in our study, may prove to be the most convenient method. Following further investigation into its potential, it could be established as the main method in histological procedures to facilitate decision-making as to the most efficacious medication.

## Abbreviations

DG: Densely granulated
hGH: Human growth hormone
IGF-1: Insulin-like growth factor-1
IHC: Immunohistochemistry
IRS: Immunoreactive score
LA-SSAs: Long-acting somatostatin analogs
PRL: Prolactin
SA: Somatostatin analog
SG: Sparsely granulated
SRL: Somatostatin receptor ligands
SST: Somatostatin
SSTR: Somatostatin receptor
STH: Somatotrophic hormone
TSS: Transsphenoidal surgery
ULN: Upper limit of normal
